# Role of precursors mixing sequence on the properties of CoMn_2_O_4_ cathode materials and their application in pseudocapacitor

**DOI:** 10.1038/s41598-019-53364-2

**Published:** 2019-11-14

**Authors:** Bhaskar Pattanayak, Firman Mangasa Simanjuntak, Debashis Panda, Chih - Chieh Yang, Amit Kumar, Phuoc – Anh Le, Kung – Hwa Wei, Tseung –Yuen Tseng

**Affiliations:** 1 Department of Electrical Engineering and Computer Science, National Chiao Tung University, Hsinchu, 30010 Taiwan; 2 Institute of Electronics, National Chiao Tung University, Hsinchu, 30010 Taiwan; 3 WPI-Advanced Institute for Materials Research, Tohoku University, Sendai, 980-8577 Japan; 4 Department of Physics, National Institute of Science and Technology, Berhampur, Orissa 761008 India; 5 Department of Materials Science and Engineering, National Chiao Tung University, Hsinchu, 30010 Taiwan

**Keywords:** Materials science, Materials for energy and catalysis, Batteries

## Abstract

In this study, the effect of oxygen vacancy in the CoMn_2_O_4_ on pseudocapacitive characteristics was examined, and two tetragonal CoMn_2_O_4_ spinel compounds with different oxygen vacancy concentrations and morphologies were synthesized by controlling the mixing sequence of the Co and Mn precursors. The mixing sequence was changed; thus, morphologies were changed from spherical nanoparticles to nanoflakes and oxygen vacancies were increased. Electrochemical studies have revealed that tetragonal CoMn_2_O_4_ spinels with a higher number of oxygen vacancies exhibit a higher specific capacitance of 1709 F g^−1^ than those with a lower number of oxygen vacancies, which have a higher specific capacitance of 990 F g^−1^. Oxygen vacancies create an active site for oxygen ion intercalation. Therefore, oxidation–reduction reactions occur because of the diffusion of oxygen ions at octahedral/tetrahedral crystal edges. The solid-state asymmetric pseudocapacitor exhibits a maximum energy density of 32 Wh-kg^−1^ and an excellent cyclic stability of nearly 100%.

## Introduction

Energy storage components have received considerable attention in the design of small microelectronic devices. Pseudocapacitors are essential devices for energy storage applications because of their high power densities, rapid charge–discharge rate, and long lifecycle^1^. For renewable and sustainable energy applications, the adoption of new nanostructured materials is crucial for manufacturing hybrid supercapacitors^2^. Metal oxides with a spinel crystal structure of AB_2_X_4_, where A and B are metal and X is oxygen, are preferred materials for manufacturing hybrid supercapacitors because of their larger specific capacitances and superior energy storage ability compared with carbon-based supercapacitors^3,4^. Among various types of spinel structures, cobalt manganese oxide (i.e., Co_3−x_Mn_x_O_4_) has distinct characteristics and excellent advantages in the field of charge storage devices. The presence of multiple valences of cations (Co and Mn ions) demonstrates excellent electrochemical behaviour. However, the effect of oxygen vacancy defects in Co_3−x_Mn_x_O_4_-based pseudocapacitor devices has not yet been investigated.

Various synthesised methods have been proposed for producing spinel-structured materials, such as solid-state reactions^3,5^, the sol–gel^6^ method, the hydrothermal method^7^, and the co-precipitation method^8^. However, most methods require high temperatures and considerable amounts of time, which hinder their applications. Because high-temperature processing engenders irregular structures, with a low surface area and large particle size, it is associated with unsatisfactory chemical properties^7–9^.

In this study, an air oxidation precipitation method was adopted to synthesise the tetragonal CoMn_2_O_4_ spinel oxides, where the crystalline spinel was fabricated at a lower temperature and in less time than those in other studies. High-temperature annealing in air and argon atmospheres have been reported to successfully generate oxygen vacancies^10,11^. Furthermore, the solvothermal method can generate oxygen vacancies but requires a long period of time^12^. This method allowed us to modulate the oxygen vacancy concentration in CoMn_2_O_4_ by merely varying the precursor mixing sequence without changing precursor ingredients. Therefore, structural and morphological studies were conducted using X-ray diffraction (XRD), scanning electron microscopy (SEM), and transmission electron microscopy (TEM) analyses. The effect of oxygen vacancies on the energy storage behaviour of cathode materials was investigated using X-ray photoelectron spectroscopy (XPS).

## Results and Discussion

**Figure 1:**
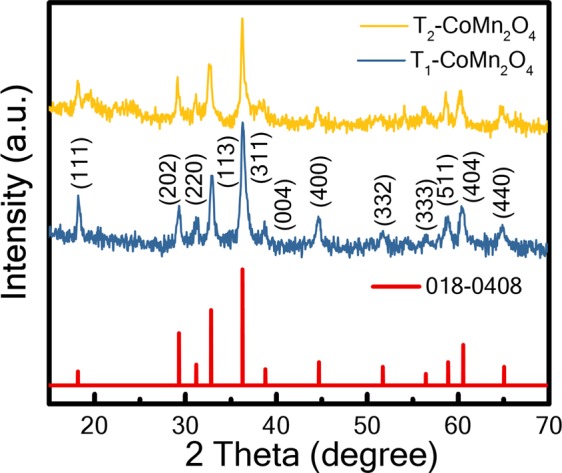
(**a**) X-ray diffraction spectra of synthesised T_1_-CoMn_2_O_4_ and T_2_-CoMn_2_O_4_. Experimental data with Bragg diffraction position are marked by a blue line (T_1_-CoMn_2_O_4_), a yellow line (T_2_-CoMn_2_O_4_), and red bars.

Figure 1 shows the XRD spectra of T_1_ and T_2_ samples. The obtained Bragg’s diffraction patterns were matched with the standard JCPDS data file (no #18-0408), which validated the formation of the tetragonal structure of CoMn_2_O_4_ with a mixed spinel (Co, Mn) (Co, Mn)_2_O_4_^11^. The strong peaks in the spectra indicated that the as-synthesised T_1_ and T_2_ had satisfactory crystallinity.

**Figure 2:**
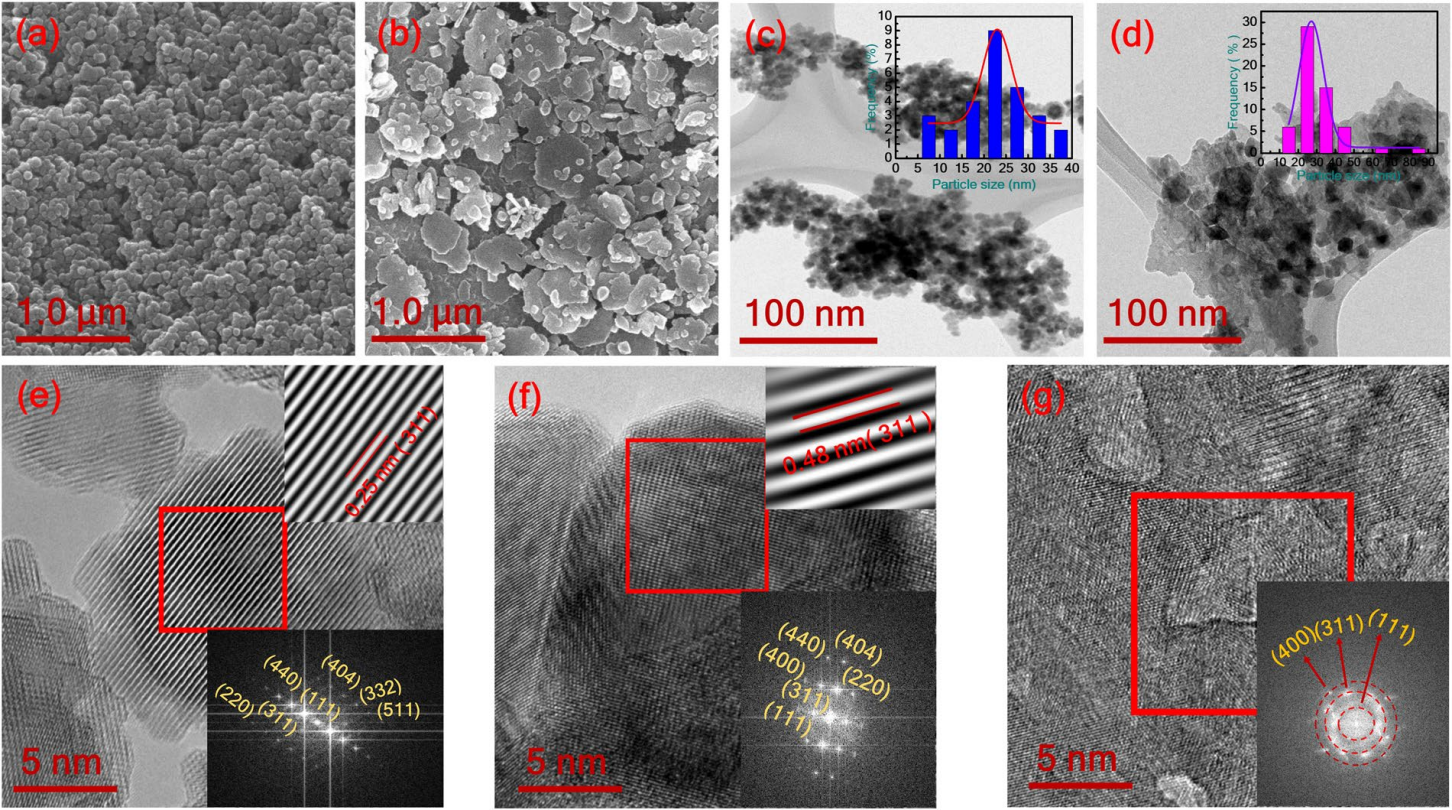
(**a**,**b**) Scanning electron microscopy images of T_1_-CoMn_2_O_4_ and T_2_-CoMn_2_O_4_ and (**c**,**d**) transmission electron microscopy images of T_1_-CoMn_2_O_4_ and T_2_-CoMn_2_O_4_ with particle size distribution (inset). High-resolution transmission electron microscopy images and the corresponding fast Fourier transform of (**e**) T_1_-CoMn_2_O_4_ and (**f**,**g**) T_2_-CoMn_2_O_4_.

Figure 2 shows the SEM and TEM micrographs of T_1_ and T_2_. Figure 2(a,b) show SEM images of T_1_ and T_2_, respectively, which indicate that T_1_ comprises uniformly distributed spherical nanoparticles, whereas T_2_ primarily comprises nanoflakes and a few nanoparticles with a square structure. Figure 2(c,d) illustrate TEM images of T_1_ and T_2_, respectively, and T_1_ and T_2_ nanoparticles are aggregated with an average particle size of approximately 23 and 27 nm, respectively [insets of Fig. 2(c,d)]. Moreover, similar nanoparticle sizes were reported in spinel CoMn_2_O_4_ nanoparticles supported on nitrogen phosphorus-doped graphene electrode materials by He’s group, in which the range of particle sizes was 5–25 nm^13^. Therefore, T_2_ has higher conductivity than T_1_, which is validated by electrochemical impedance spectroscopy (EIS). To identify the phase of the samples, high-resolution transmission electron microscopy was performed on T_1_ and T_2_ nanostructures [as shown in Fig. 2(e–g)], respectively. The selected area electron diffraction (SAED) patterns of T_1_ and T_2_ reveal crystal diffraction with planes of (220), (311), (440), (111), (404), (332), and (511) for T_1_ and T_2_ [insets of Fig. 2(e–g)], which validate the formation of the tetragonal spinel crystal structure. The lattice fringes of T_1_ nanoparticles show (311) plane orientation with interplanar spacing of 2.5 Å, and those of T_2_ samples show (111) plane orientation with interplanar spacing of 4.8 Å [insets of Fig. 2(e,f)]. However, nanoflakes exhibit lattice fringes of (400), (311), and (111) for T_2_ [Fig. 2(g)]. This result was consistent with XRD results (Fig. 1).

**Figure 3:**
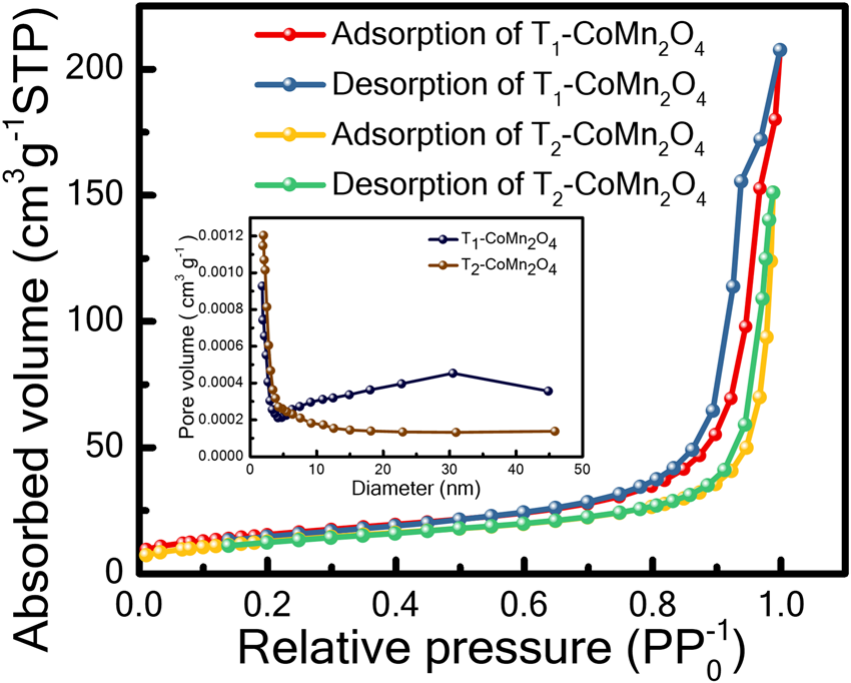
Brunauer–Emmett–Teller surface area of T_1_-CoMn_2_O_4_ and T_1_-CoMn_2_O_4_; inset presents pore-size distribution.

The surface area and porosity of the electrodes are crucial in the performance of pseudocapacitor devices^14,15^; therefore, a Brunauer–Emmett–Teller (BET) surface area measurement was performed. Figure 3 shows nitrogen adsorption–desorption isotherms at 77 K of T_1_ and T_2_. According to the definite hysteresis loop of IUPAC classification, the isotherms of both spinels are classified as type IV, which corresponds to the mesoporous structure^13,16,17^. The availability of hysteresis loop intermediate P/P_0_ = 0.5 to 1 confirms the mesoporous nature of both compounds^15^. A high adsorption P/P_0_ at 0.9 to 1 indicates the performance of the macroporous part to the overall surface area of T_1_ and T_2_^14^. The BET surface areas and the corresponding pore volumes of T_1_ and T_2_ are 53.21 m²/g (0.32 cm³/g) and 44.47 m²/g (0.23 cm³/g), respectively. A higher surface area corresponds to a smaller grain size^18^. The pore-size distribution curve can be obtained through N_2_ adsorption–desorption isotherms by using the Barrett–Joyner–Halenda (BJH) method (inset of Fig. 3). The average pore sizes of T_1_ and T_2_ are 20.0 and 20.8 nm, respectively.

**Figure 4:**
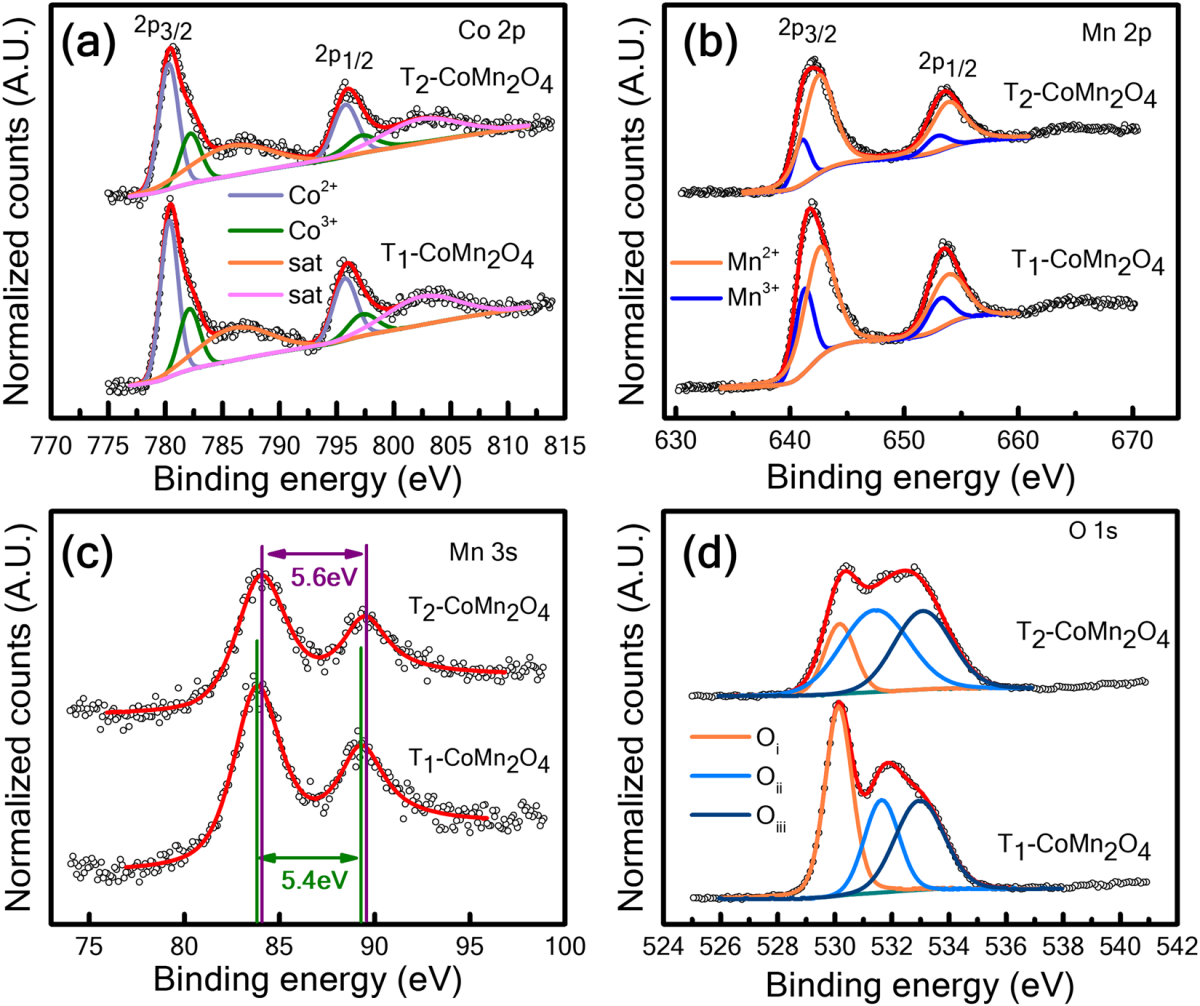
X-ray photoelectron spectra of the synthesised spinel (**a**) Co 2p of T_1_-CoMn_2_O_4_ and T_2_-CoMn_2_O_4_, respectively; (**b**,**c**) Mn 2p and Mn 3 s of T_1_-CoMn_2_O_4_ and T_2_-CoMn_2_O_4_, respectively; (**d**) O 1 s spectra of T_1_-CoMn_2_O_4_ and T_2_-CoMn_2_O_4_, respectively.

An XPS analysis was conducted to evaluate defects and oxidation states in the CoMn_2_O_4_ spinel structure. Figure 4 shows the XPS spectra of Co2p, Mn2p, Mn3s, and O1s core levels of pure T_1_ and T_2_ samples. Figure 4(a) presents the Co2p spectra of T_1_ and T_2_, respectively, indicating that there are doublet peaks of Co 2p_3/2_ and Co 2p_1/2_ located at 780.46 and 795.96 eV for T_1_ and 780.62 and 796 eV for T_2_, respectively. The energy splitting (ΔE) values of the divalent and trivalent Co ions are 15.4 and 15.1 eV for T_1_ and T_2_, respectively, which are consistent with values reported in the literature^19^. The Co2p spectra of T_1_ and T_2_ are fitted into six peaks with binding energies of approximately 780.4 and 795.8 eV for Co^2+^ and 782.2 and 797.3 eV for Co^3**+**^ ^5,19^. Furthermore, two satellite peaks are observed at 786.13 and 802.6 eV for T_1_ and 785.84 and 802.45 eV for T_2_, respectively^19^. The concentrations of Co^2+^ and Co^3+^ are 63% and 37% for T_1_ and 70% and 30% for T_2_, respectively(as listed in Table 1).

**Table 1:** Various valence numbers of ions in T_1_ and T_2_ from X-ray photoelectron spectroscopy data.

Valance states	T_1_-CoMn_2_O_4_ (%)	T_2_-CoMn_2_O_4_ (%)
Co^3+^	37	30
Co^2+^	63	70
Mn^2+^	73	82
Mn^3+^	27	18

Pattanayak *et al*., Table 1.

Figure 4(b,c) show the Mn 2p and Mn 3 s spectra of T_1_ and T_2_, respectively. Figure 4(b) shows the Mn 2p spectra combined with 2p_3/2_ and 2p_1/2_ located at approximately 641.9 and 653.8 eV for T_1_ and 642 and 653.7 eV for T_2_, respectively. The Mn2p_3/2_ and Mn2p_1/2_ of T_1_ are deconvoluted into sub-peaks located at approximately 641.39 and 652.91 eV for Mn^3+^ and 642.5 and 654 for Mn^2+^ states, respectively. Furthermore, those of the T_2_ sample are deconvoluted into sub-peaks located at approximately 641.2 and 652.91 eV corresponding to Mn^3+^ and 642.5 and 653.9 eV for Mn^2+^ states^20^. The concentrations of Mn^2+^ and Mn^3+^ in T_1_ are 73% and 27%, respectively, whereas those for T_2_ are 82% and 18%, respectively (Table 1). To ensure peak splitting and doublet, the parallel spin coupling of Mn 3 s of T_1_ and T_2_ were analysed, and Fig. 4(c) depicts the results. Mn 3 s core level spectra show binding energies at approximately 83.9 and 89.3 eV for T_1_ and 83.9 and 89.5 eV for T_2_. The energy separation for T_1_ is 5.4 eV, which is lower than that for T_2_, (5.6 eV) [Fig. 4(c)], indicating the dominance of lower Mn valance^10,21–23^. Figure 4(d) shows the peak fitting for the O1s core level of both T_1_ and T_2_ indicating that three peaks are observed at approximately 530.16 (O_i_), 531.67 (O_ii_), and 533 eV (O_iii_) for T_1_ and 530.2 (O_i_), 531.49 (O_ii_), and 533.14 eV (O_iii_) for T_2_. The O_i_, O_ii_, and O_iii_ correspond to lattice oxygen bonding with metal (Co, Mn) O_metal-oxygen_, non-lattice oxygen (oxygen vacancies), and oxygen absorbed on the surface in the form of OH^−^, respectively. Figure 4(d) presents that the large area covered by the O_ii_ peak indicates the presence of higher oxygen vacancies in T_2_^11,24^. The percentages of oxygen vacancies in T_1_ and T_2_ samples are 35% and 72%, respectively.

**Figure 5:**
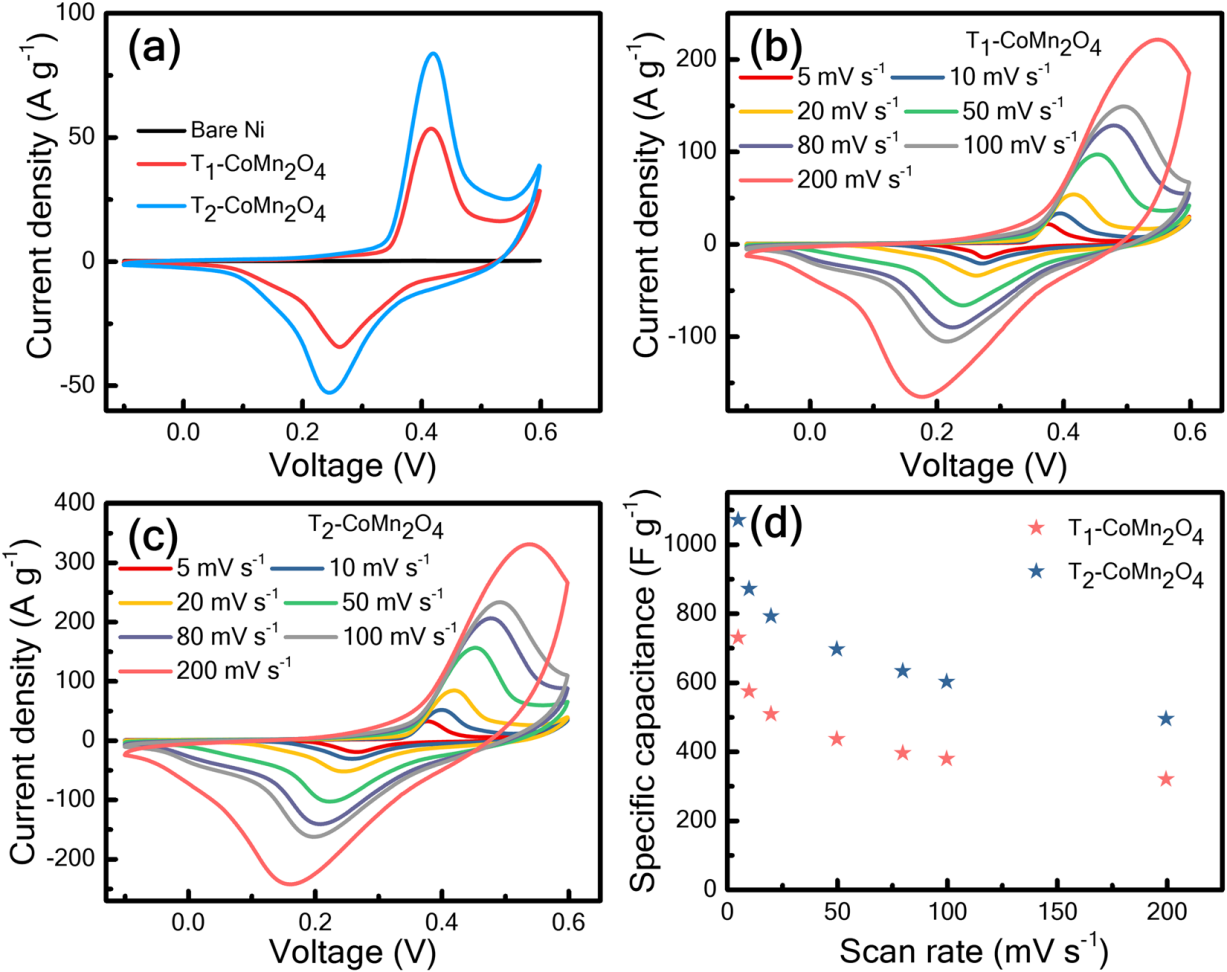
Electrochemical characterisation of spinel material electrodes: (**a**) the cyclic voltammetry responses of bare Ni foam T_1_-CoMn_2_O_4_ and T_2_-CoMn_2_O_4_, respectively, at 5 mV s^−1^; (**b**,**c**) cyclic voltammetry responses of T_1_-CoMn_2_O_4_ and T_2_-CoMn_2_O_4_, respectively, at a scan rate of 5–200 mV s^−1^; (**d**) variation in the specific capacitance with the scan rate for two electrodes.

Electrochemical performance examinations of T_1_ and T_2_ electrodes were conducted using a three-electrode cell system. The cyclic voltammetry (CV) responses of T_1_, T_2_, and bare Ni foam are presented in Fig. 5(a) at a scan rate of 20 mV s^−1^ in a voltage window from −0.1 to 0.6 V and indicate that both T_1_ and T_2_ exhibit oxidation and reduction redox peaks at approximately 0.2 and 0.5 V, respectively, because of the Faradic reaction. The quasi-reversible Faradic reaction with redox peaks for T_1_ and T_2_ reveals the occurrence of surface redox reactions, which are provided as follows^25,26^.

$$CoM{n}_{2}{O}_{4}+O{H}^{-}+{H}_{2}O\rightleftarrows CoOOH+MnOOH+{e}^{-}$$

$$CoOOH+O{H}^{-}\rightleftarrows Co{O}_{2}+{H}_{2}O+{e}^{-}$$

$$MnOOH+O{H}^{-}\rightleftarrows Mn{O}_{2}+{H}_{2}O+{e}^{-}$$

Figure 5(b,c) show the CV responses of T_1_ and T_2_ at a scan rate from 5 to 200 mV s^−1^ at −0.1 to 0.6 V, respectively. Moreover, no significant changes are observed in the shape of CV curves when the scan rate increased from 5 to 200 mV s^−1^, indicating that both electrodes have satisfactory electronic conduction and low equivalent series resistance^27^. Because of the kinetics of redox reactions, the current increases with the increasing scan rate in accordance with Ohm’s law^26^. Because of the internal resistance between the electrode and electrolyte ions, the anodic peaks show red shift of 0.17 V and cathodic peaks blue shift of 0.1 V with the increasing scan rate for T_1_ which is higher than T_2_ (0.16 V for the anodic scan and 0.1 V for the cathodic scan) [Fig. 5(b,c)]^27,28^. The reduced peak shifting of T_2_ indicates that a lower overpotential is required for ionic transport because of a high current response at higher scan rates. Therefore, T_2_ shows a higher rate capability than T_1_.

Specific capacitance (F g^−1^) can be estimated from the CV curve by using the following equation^14,18^:

1 $${C}_{sp}=\frac{{\int }_{{V}_{1}}^{{V}_{2}}I(V)dV}{m\gamma ({V}_{2}-{V}_{1}\,)}$$

where γ, m, (V_2_ − V_1_), and I represent the scan rate, mass of active materials, voltage window, and current response, respectively. Figure 5(b,c) show that the specific capacitances (C_sp_) of T_1_ and T_2_ calculated using Eq. (1) are 730 and 1071 F g^−1^ at 5 mV s^−1^, respectively. The scan rate dependence C_sp_ [Fig. 5(d)] indicates that C_sp_ values of T_1_ and T_2_ decrease with the increasing scan rate, which is attributed to the decreasing redox active sites with an electrolyte at high scan rates^14,29^. The mesoporous structure in the electrodes creates low-resistance pathways for ion diffusion and increased charge transport. Therefore, electrolyte ions are soaked by the mesoporous wall and increase the capacity of the electroactive channel for high charge storage at a high scan rate^16^. The area under the CV curve of T_2_ is larger than that of T_1_, thereby improving capacitive performance and ionic conductivity. Because both T_1_ and T_2_ electrodes are constructed using nanocrystalline materials with a mesoporous structure, electrolyte ions could be transported through their nanochannels^14^. When the scan rate increased to 200 mV s^−1^, T_1_ and T_2_ show specific capacitance of 318 and 493 F g^−1^ with 43% and 46% retention of the initial capacitances, respectively. The variation in charge storage efficiency may be attributed to the difference between the crystallite size and surface morphology of T_1_ and T_2_.

**Figure 6:**
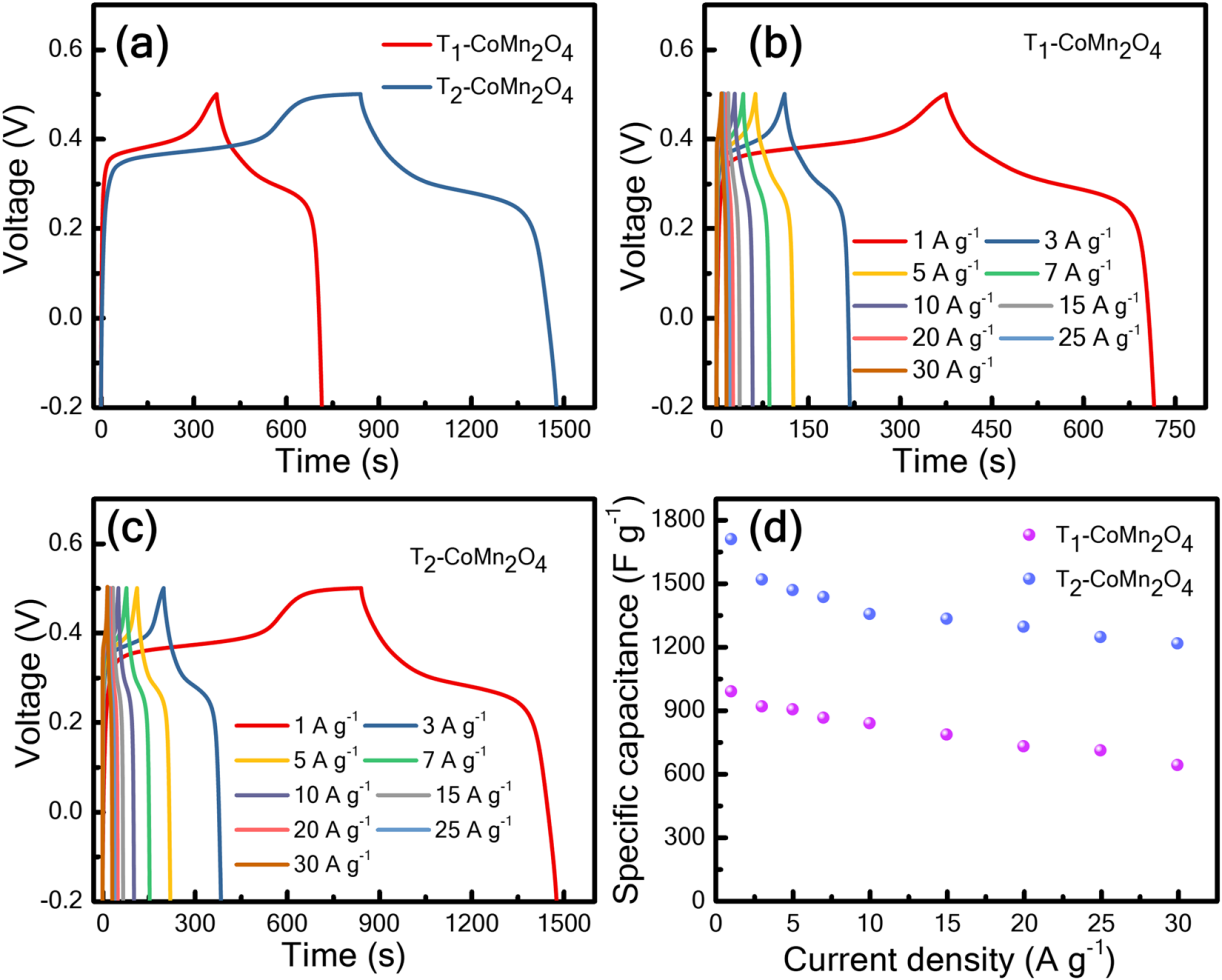
(**a**) Galvanostatic charge–discharge profiles of T_1_-CoMn_2_O_4_ and T_2_-CoMn_2_O_4_ electrodes, respectively, at a current density 1 A g^−1^; (**b**,**c**) charge–discharge responses of T_1_-CoMn_2_O_4_ and T_2_-CoMn_2_O_4_ with current densities ranging from 1 to 30 A g^−1^, respectively; (**c**) variation in specific capacitance with current density of T_1_-CoMn_2_O_4_ and T_2_-CoMn_2_O_4_.

CV performance was significant for the infliction of potential in pseudocapacitive devices. Furthermore, charge–discharge behaviour was one of the most crucial characteristics. Figure 6(a) exhibits that the charge–discharge characteristics of T_1_ and T_2_ are −0.2 to 0.5 V at a current density of 1 A g^−1^, indicating that the discharge profile is nonlinear, which is prominent pseudocapacitance within this potential window. These discharge profiles can be divided into two parts. One part varies nonlinearly from 0.5 to approximately 0.25 V, suggesting pseudocapacitor behaviour, whereas the other parameter varies from approximately 0.25 to −0.2 V, indicating a double-layer capacitor mechanism^14^. This phenomenon is attributed to the quasi-reversible redox reaction at the electrode–electrolyte interface^2,14^. Figure 6(a,b) show the charge–discharge profiles of T_1_ and T_2_ from 1 to 30 A g^−1^, which demonstrate Faraday pseudocapacity with a negligible voltage drop.

The specific capacitance (F g^−1^) can be calculated from the charge–discharge curve as follows^30^:

2 $${C}_{sp}=\frac{2\times I\times {\int }_{t1}^{t2}Vdt}{m\times ({V}_{2}^{2}-{V}_{1}^{2})}$$

where m, I, V_2_, V_1_, and ${\int }_{{t}_{1}}^{{t}_{2}}Vdt$ represent the mass of an active material, discharge current, upper voltage, lower voltage, and area covered by the discharge curve, respectively.

Moreover, C_sp_ at current densities ranging from 1 to 30 A g^−1^ is estimated using Eq. (2), and Fig. 6(d) displays the results. Figure 6(a) reveals that the discharge time of T_2_ is considerably longer than that of T_1_ because of the longer time for the redox reaction; therefore, C_sp_ of T_2_ (1709 F g^−1^) is considerably higher than that of T_1_ (990 Fg^−1^) at 1 A g^−1^ [Fig. 6(d)]. When the current density increased to 30 A g^−1^, C_sp_ values of T_2_ and T_1_ are 1216 and 641 F g^−1^ with capacitive retention of 71% and 64%, respectively. Such high capacitive retention at high current density indicates the effect of oxygen vacancies^5^. The improved performance of T_2_ is attributed to the effective sharing of oxygen vacancies causing a high degree of contact of OH^−^ ions for Faradic reactions^31^. Note that the specific surface areas of T_1_ and T_2_ are nearly equal, yet, the pseudocapacitive charge storage between the two materials are distinctive; therefore, we can infer that the higher C_sp_ and retention of T_2_ than those of T_1_ are due to the higher oxygen vacancies concentration in T_2_^32^_,_ as discussed below. In the CV procedure, the current response is measured with respect to voltage at constant time. However, galvanostatic charge–discharge (GCD) measurement is performed at a constant current for a varying voltage with respect to time. GCD measurement is generally more applicable to the study of the pesudocapacitive material^33^. A slightly higher specific capacitance is observed in this study for the GCD method compared with CV [Figs 5(d) and 6(d), respectively], which is attributed to the different measurement procedures^33^.

**Figure 7:**
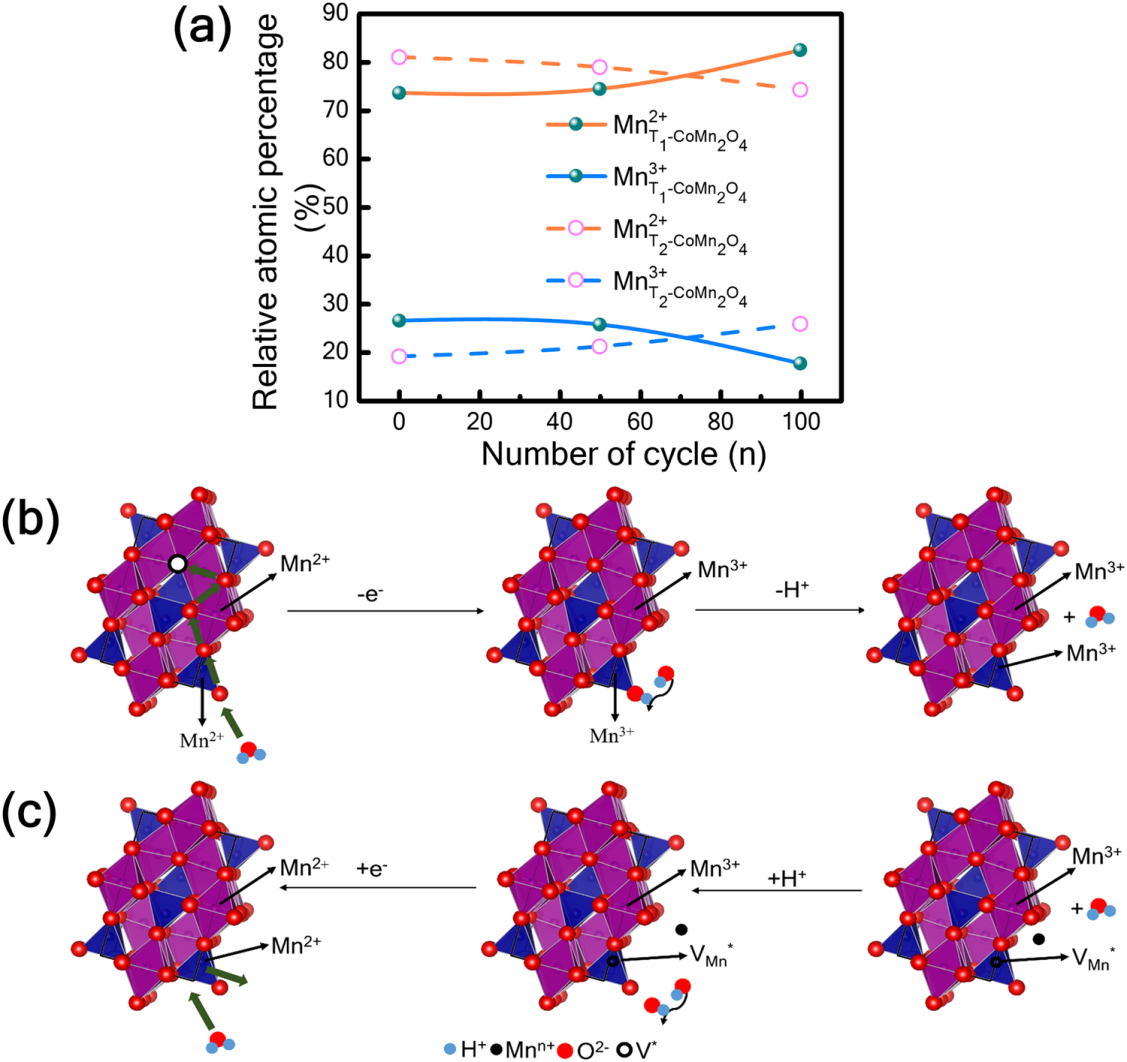
(**a**) Variation in manganese oxidation states in T_2_ cathode after 50 and 100 cycles according to X-ray photoelectron spectroscopy and (**b**,**c**) mechanism of oxygen intercalation into T_2_-CoMn_2_O_4_ cathode.

To understand the redox nature of oxygen intercalation in CoMn_2_O_4_, *ex-situ* XPS of T_1_ and T_2_ cathode materials along with carbon black are also performed after 50 and 100 consecutive CV cycles (the results shown in the Supplementary Information, Figs S1–S3). Figure 7(a) shows different manganese oxidation states in the T_1_ or T_2_ cathode material evaluated after 50 and 100 CV cycles. For the cathode T_1_, atomic concentrations of Mn^3+^ and Mn^2+^ remain unchanged up to 50 cycles. After 100 CV cycles, interestingly, the atomic % of Mn^3+^ and Mn^2+^ significantly change. Mn^3+^ decreases from 26 to 18 while Mn^2+^ increases from 74 to 82. As for the cathode T_2_, Mn^3+^ slightly increases from 19 to 21 atomic % and Mn^2+^ decreases from 81 to 79 atomic % when changing to 50 cycles. After 100 cycles, Mn^3+^ reaches an atomic % of 26 and Mn^2+^ has 74. Therefore, the net 8% decrease of Mn^3+^ for T_1_ while 7% increase for T_2_ after 100 CV cycles are found. In the meantime, it is shown in Fig. 7(a) 8% increase of Mn^2+^ for T_1_ and 7% decrease for T_2_ after 100 cycles. On the other hand, under consideration of oxygen in the present cathode material, the O^2−^ ions from the electrolyte should have sufficient resistance to being inserted into a densely packed structure of the cathode material at room temperature to fill the oxygen vacancies without external field^32^. In addition, OH^−1^ ion from the electrolyte(0.5 M LiOH aqueous solution) would be absorbed by oxygen vacancy site leading to transfer its proton to a neighbouring lattice oxide after applied voltage. As a result, oxygen vacancy would be filled after 50 CV cycles. In general, the valence change of transition metal oxide(such as Mn), (Fig. 7(a)) is correlated with the concentration of charged oxygen vacancy, where atomic concentration of Mn^3+^ continuously increases while that of Mn^2+^ decreases up to 100 cycles in T_2_ cathode material. In the mean time, the valance change in Co of T_2_ cathode is not obvious(Supplemental Fig. S3(c)). In this case, the concentration of charged oxygen vacancy would be decreased. But based on Supplemental Fig. S2(c)), the concentration of oxygen vacancy gradually increases up to 100 cycles for both T_1_and T_2_ cathode materials. Therefore, it seems contradiction in this case. This phenomenon may probably be attributed to the valence change of charged oxygen vacancy, complex reactions between electrolyte and composite electrode, and/or that the O1s peak contains some additional low intensity peak related to carbon material (example C=O, C-O, etc.) in the cathode affected the vacancy calculation. It needs to be proved in the future work. Figure 7(b,c) show the mechanism for the T_2_ cathode material. Both T_1_ and T_2_ comprise multivalence Mn in lattice sites, primarily Mn^2+^ and Mn^3+^ [Fig. 4(b,c)], suggesting the electron transition occurred from Mn^2+^  → Mn^3+^ and vice versa. Through intercalation, oxygen vacancies in T_1_ and T_2_ cathode material are combined with oxygen, thereby altering the oxidation states of manganese. Oxygen ions are diffused, resulting in the oxidation state of Mn^2+^ to Mn^3+^, which is validated in Fig. 7(a). Therefore, it is concluded that material T_2_ has almost equal surface area and more oxygen vacancies than T_1_; however, T_2_ has higher capacitance, Its charge storage is believed to be due to oxygen intercalation^32^. The cobalt oxidation states of  T_1_ and T_2_ cathode materials are almost stable after various CV cycles. This indicates that the manganese is crucial for charge storage (Supplemental Information S3).

**Figure 8:**
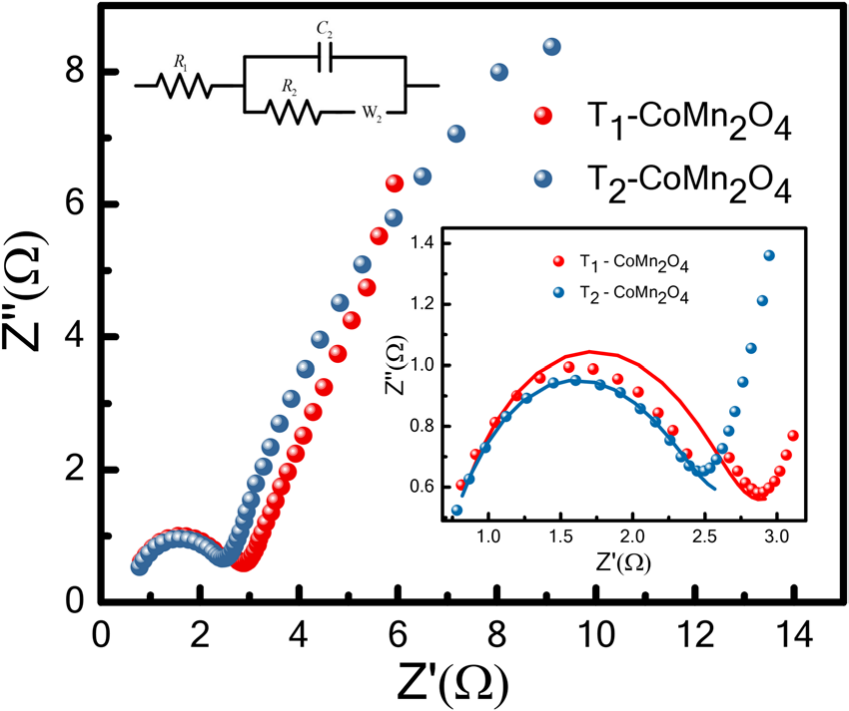
Electrochemical impedance spectroscopy impedance characteristics of T_1_-CoMn_2_O_4_ and T_2_-CoMn_2_O_4_; inset presents the high-frequency region with an equivalent circuit of T_1_-CoMn_2_O_4_ and T_2_-CoMn_2_O_4_.

Because of the prevalence of the electron transport and kinetic features of ions at the electrode–electrolyte interface, the pseudocapacitor exhibited a low internal resistance^14,34^. To understand this phenomenon, EIS measurements are performed at frequencies ranging from 1 Hz to 1 MHz. Figure 8 displays the conventional impedance spectra of T_1_ and T_2_ electrodes. The impedance behaviour introduces a semicircle in the high-frequency region and an inclined line in the low-frequency region, thus revealing pseudocapacitor behaviour^34,35^. The intercept along Z’ represents the internal resistance of electrode R_1_. The diameter of the semicircle provides charge transfer resistance R_2_. All resistances (i.e., R_1_ and R_2_) of T_1_ are slightly higher than those of T_2_. The oxygen vacancies facilitate the migration of electrolyte ions in the conducting path^11,31,36^,. Moreover, the electron conductivity of the electrode increases with the increasing number of oxygen vacancies^11,37^. Because the concentration of oxygen vacancies is higher in T_2_ than in T_1_, the R_1_ and R_2_ values of T_2_ are 0.62 and 2.57 Ω, whereas those of T_1_ are higher, 0.65 and 2.87 Ω, respectively. The Density Functional Theory method is applied to study the effect of oxygen vacancies on the catalytic performance of manganese oxide. Oxygen vacancies increase the conductivity because of compression of the band gap^36^. The R values of T_2_ are lower than those of T_1_ and are consistent with those in this study^36^.

**Figure 9:**
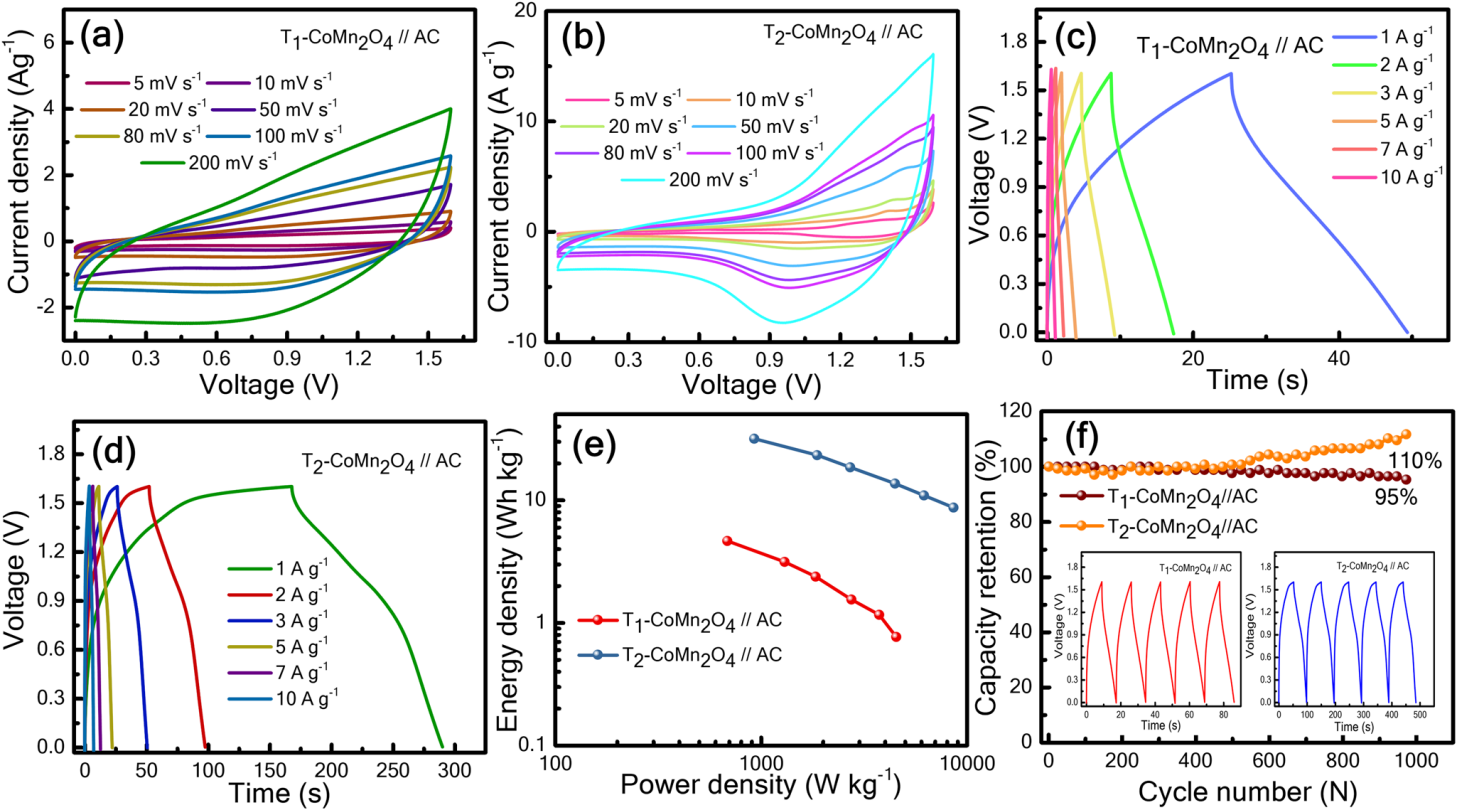
Electrochemical performance of the asymmetric psudocapacitor device: (**a**,**b**) cyclic voltammetry responses of the asymmetric device containing T_1_-CoMn_2_O_4_//AC and T_2_-CoMn_2_O_4_//AC_,_ respectively, at scan rates ranging from 5–200 mV s^−1^; (**c**,**d**) galvanostatic charge–discharge profiles of T_1_-CoMn_2_O_4_//AC and T_2_-CoMn_2_O_4_//AC, respectively, with current densities from 1–10 A g^−1^; (**e**) Ragone plots of power density versus energy density for T_1_-CoMn_2_O_4_//AC and T_2_-CoMn_2_O_4_//AC asymmetric pseudocapacitor devices; (**f**) cycle performance of T_1_-CoMn_2_O_4_//AC and T_2_-CoMn_2_O_4_//AC asymmetric pseudocapacitors with a discharge current density of 2 A g^−1^. The inset presents the galvanostatic charge–discharge curve of the asymmetric pseudocapacitor device.

Asymmetric pseudocapacitors, including T_1_//AC and T_2_//AC, are fabricated, where T_1_ and T_2_ are the cathode and commercial AC is anode material. Figure 9(a)and (b) present the conventional CV curves of T_1_ and T_2_ cells at various scan rates in the voltage ranges of 0–1.6 V, which display a nearly rectangular shape. According to the CV characteristics of the T_1_ cell [Fig. 9(a)], no redox peak is observed in a wide voltage window, whereas redox peaks are observed in the T_2_ cells at approximately 1.5 and 1.0 V [Fig. 9(b)], indicating that T_2_ cell exhibits two different energy storage mechanisms because of rapid electrolyte ion transport and redox reaction kinetics. Figure 9(c,d) represent the charge–discharge behaviours of T_1_ and T_2_ cells, respectively, at a current density of 1–10 A g^−1^. T_1_ cell shows an approximately linear charge–discharge behaviour, whereas T_2_ cell exhibits nonlinear behaviour, indicating pseudocapacitive characteristics.

Energy density E (Wh-kg^−1^) and power density P (W-kg^−1^) values are calculated using Eqs (3) and (4)^18^:

3 $$E=\frac{1}{(2\times 3.6\,)}{C}_{sp}\times {(\Delta V)}^{2}$$

4 $$P=\frac{E}{\Delta t}\times 3600$$

where C_sp_, ΔV, and Δt are specific capacitance (F g^−1^), potential window, and discharge time (s), respectively.

Figure 9(e) depicts the Ragone plots of the as-fabricated T_1_ and T_2_ cells, respectively. The energy density of T_2_ cell decreases from 32 to 9 Wh-kg^−1^ with an increase in power density from 0.9 to 8.7 kW-kg^−1^, whereas for T_1_ cell, the energy density decreases from 5 to 0.8 Wh-kg^−1^ when increasing power density from 0.7 to 5 kW-kg^−1^. T_2_ exhibits higher energy density and power density than T_1_. For achieving high-performance pseudocapacitors, obtaining high energy density and power density are crucial. The flake-like morphological structure [Fig. 2(b)] can improve diffusion paths for electrons and ions in the oxide materials and increase interfacial redox reactions, resulting in the increase in capacitance. Therefore, the T_2_//AC device exhibited high energy and power density [Fig. 9(e)]. It is believed that the mesoporous structure and flake-like morphology increase the specific capacitance, thus increasing the energy and power density of the asymmetric pseudocapacitor, T_2_. Figure 9(f) presents the cyclic stabilities of T_1_ and T_2_ cells, which indicate that the T_1_//AC exhibits the negligible decay of capacitance (95%), whereas the T_2_//AC shows 110% retention after 1000 cycles in the PVA-LiOH polymer gel electrolyte at 2 A g^−1^. This observation indicates the excellent electrochemical stability of T_1_ and T_2_ cells. The slight decay of cyclic stability for T_1_//AC is possibly attributed to the decrease in the adhesion of active materials with the current collector^27^. These results reveal that cobalt manganese oxide is a prominent electrode candidate material for making asymmetric pseudocapacitors.

## Conclusions

Modulation of the intrinsic defect concentration in CoMn_2_O_4_ tetragonal spinel was synthesised at moderate temperatures by adjusting the mixing sequence of Co and Mn precursors. Moreover, two distinct morphologies of CoMn_2_O_4_ were observed as a result of this adjustment. CoMn_2_O_4_ synthesised by mixing the Mn precursor after adding the Co precursor (T_1_) formed nanoparticle morphology. Moreover, a nanoparticle and nanoflake combined structure was formed when the Mn precursor was mixed before adding the Co precursor (T_2_). The electrochemical evaluations revealed that the T_2_ electrode had a higher specific capacitance (1709 F g^−1^) than the T_1_ electrode (990 F g^−1^) at 1 A g^−1^ with capacitive retentions of 71% and 64% for T_2_ and T_1_, respectively. A higher number of oxygen vacancies existed in the T_2_ electrode, which enhanced the capacitance because of the intercalation of oxygen ions/oxygen vacancies from the aqueous alkaline electrolyte. The T_2_//AC asymmetric pseudocapacitor exhibited a maximum energy density of 32 Wh-kg^−1^ at a power density of 0.9 kW-kg^−1^. The T_2_//AC pseudocapacitor had a higher energy density and power density than T_1_//AC. Both T_1_ and T_2_ cells demonstrated excellent electrochemical stability. This study not only demonstrates that the mixing sequence of the precursors during CoMn_2_O_4_ synthesis is crucial in determining the performance of the pseudocapacitor but also provides insights into the mechanism of charge storage with oxygen vacancies. The present study develops a new type of CoMn_2_O_4_-based electrode for future pseudocapacitor applications.

## Methods

### Materials

To synthesise the nanocrystalline cobalt manganese oxide spinel materials, the precursor materials of cobalt nitrate hexahydrate [Co (NO_3_)_2_, 6H_2_O] (Alfa Aesar), manganese nitrate tetrahydrate [Mn(NO_3_)_2_, 4H_2_O] (Alfa Aesar), and an aqueous ammonia solution were used. All chemicals were used without any purification.

### Synthesis of the CoMn_2_O_4_ spinel structure

In accordance with a conventional process, 25 ml of 0.2 M [Co(NO_3_)_2_, 6H_2_O] was stirred at room temperature, and 20 ml of ammonia solution was slowly added to this solution. Moreover, 50 ml of 0.2 M [Mn(NO_3_)_2_, 4H_2_O] was subsequently added dropwise to the mixture and stirred for 2 h. To decompose nitrates, the mixture was vacuum-filtered and heated at 180 °C for 1 h in the air, and the sample was denoted as T_1_-CoMn_2_O_4_ (T_1_). Similarly, another sample was synthesised using the same stoichiometric amounts of Co and Mn precursors as the first sample; however, 25 ml of 0.2 M [Co(NO_3_)_2_, 6H_2_O] was added after adding [Mn(NO_3_)_2_, 4H_2_O]. The sample was denoted as T_2_-CoMn_2_O_4_ (T_2_).

### Material characterisation

To identify the crystal structure powder, XRD was conducted with Cu Kα radiation using a Bruker D2 PHASER. The specific surface area of nanomaterials was characterised by the BET surface area analyser (ASAP, 2020). SEM was conducted to study the surface microstructure (Hitachi SU-8010). A high-resolution TEM analysis was used to analyse the formation of the nanostructure and composition of materials (JEOL JEM-2010F). To examine the oxidation states of spinel oxides, XPS (ULVAC-PHI Quantera SXM) was performed.

### Preparation of working electrodes

To fabricate the working electrode, active material powders were mixed in the N-methyl-2-pyrrolidone (NMP) solution to form a uniform slurry of 10 mg/ml. A porous nickel foam substrate was washed with acetone and etched using a 6 M HCl solution for 30 min. After being washed using deionised water (DI water), the substrate was dried at 70 °C for 6 h. Next, the slurry containing active materials was coated onto the substrate (area of 1 cm^2^) using a brush and dried at 80 °C for 15 h under a vacuum condition to study its electrochemical properties and mechanisms. The mass loadings of T_1_ and T_2_ were 0.3 and 0.15 mg, respectively.

### Asymmetric pseudocapacitor assembly

The asymmetric pseudocapacitor was fabricated using activated carbon (AC) as an anode material, and T_1_ or T_2_ was used as a cathode material. The anode electrode was prepared using a mixture of AC, a polyvinylidene fluoride (PVDF) binder, and carbon black in a weight ratio of 85:10:5 in a N-methylpyrrolidone (NMP) solution to form homogeneous dispersion for deposition on the substrate. For fabricating a cathode electrode, active materials (T_1_ or T_2_), PVDF, and carbon black were used in a weight ratio of 75:10:15 and dispersed in the NMP solution. The slurries (T_1_ and T_2_) were coated on the nickel foam substrates (current collector) and dried at 80 °C for 15 h in a vacuum to form the cathodes.

To assemble pseudocapacitor devices, the solid-state electrolyte was first prepared: 1 g of PVA and 1 g of LiOH were dispersed in 20 ml of water and heated at 90 °C to form a transparent gel. After cooling, cathode and anode electrodes were then immersed on the gel electrolyte and dried at room temperature. Both electrodes were pressed to fabricate hybrid supercapacitors and were denoted as T_1_-CoMn_2_O_4_/PVA-LiOH/AC (T_1_ cell) and T_2_-CoMn_2_O_4_/PVA-LiOH/AC (T_2_ cell). For an asymmetric pseudocapacitor, the charges (Q) for cathode and anode electrodes were balanced using the following equation: Q = CmΔE, where C, m, and ΔE are the specific capacitance, mass of active materials, and potential window, respectively. After calculations, the total masses of T_1_ and T_2_ cell devices were approximately 1.5 and 0.5 mg, respectively.

### Electrochemical characterisation

The electrochemical performances of electrodes and pseudocapacitors were measured using an electrochemical analyser (Instruments CHI618B), which included CV, galvanostatic charge–discharge cycling (GCC), and EIS. Electrochemical characterisations of the electrode materials were performed using three-electrode cells with a saturated calomel electrode, platinum as a counter electrode, a working electrode of an active material deposited on the Ni foam, and 0.5 M LiOH serving as the electrolyte. The working electrode was immersed in the electrolyte for providing suitable contact between the electrode and electrolyte.

## Supplementary information

Supplementary Information

## Data Availability

Readers can access data by contacting the corresponding author.
