# Supplementary material for: Role of precursors mixing sequence on the properties of CoMn2O4 cathode materials and their application in pseudocapacitor
Source: Sci Rep. 2019 Nov 14;9:16852. doi: 10.1038/s41598-019-53364-2 (PMC6856552; doi:10.1038/s41598-019-53364-2)
Supplement: Supplementary file 1 — Supplementary Information [file 41598_2019_53364_MOESM1_ESM.pdf]

Supplementary Information

Role of precursors mixing sequence on the properties of $CoMn_2O_4$ cathode materials and their application in pseudocapacitor

Bhaskar Pattanayak[1,2], Firman Mangasa Simanjuntak[3], Debashis Panda[2,4], Chih - Chieh Yang[2], Amit Kumar[5], Phuoc – Anh Le[5], Kung – Hwa Wei[5], and Tseung –Yuen Tseng[1,2 *]

[1] Department of Electrical Engineering and Computer Science, National Chiao Tung University, Hsinchu 30010, Taiwan

[2] Institute of Electronics, National Chiao Tung University, Hsinchu 30010, Taiwan

[3] WPI-Advanced Institute for Materials Research, Tohoku University, Sendai 980-8577, Japan

[4] Department of Physics, National Institute of Science and Technology, Berhampur, Orissa, 761008, India.

[5] Department of Materials Science and Engineering, National Chiao Tung University, Hsinchu 30010, Taiwan

*tseng@cc.nctu.edu.tw

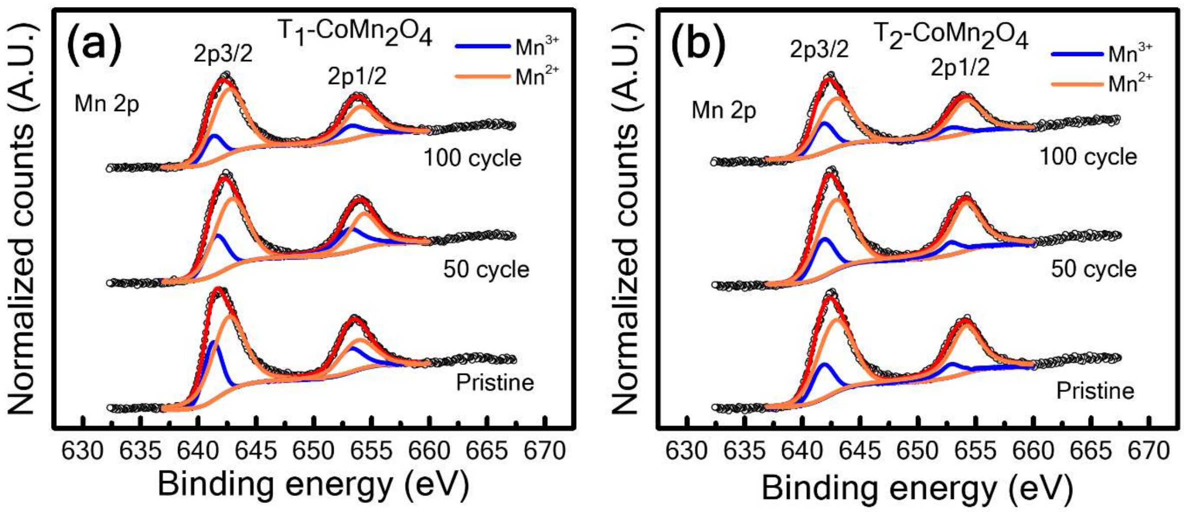

Figure S1 Ex situ XPS studies of Mn 2p of (a) T_1-$CoMn_2O_4$ and (b) T_2-$CoMn_2O_4$ in the cathodes of the supercapacitors containing active material (T_1 or T_2), conductive material (carbon black) and binder (PVDF) after different consecutive CV cycles.

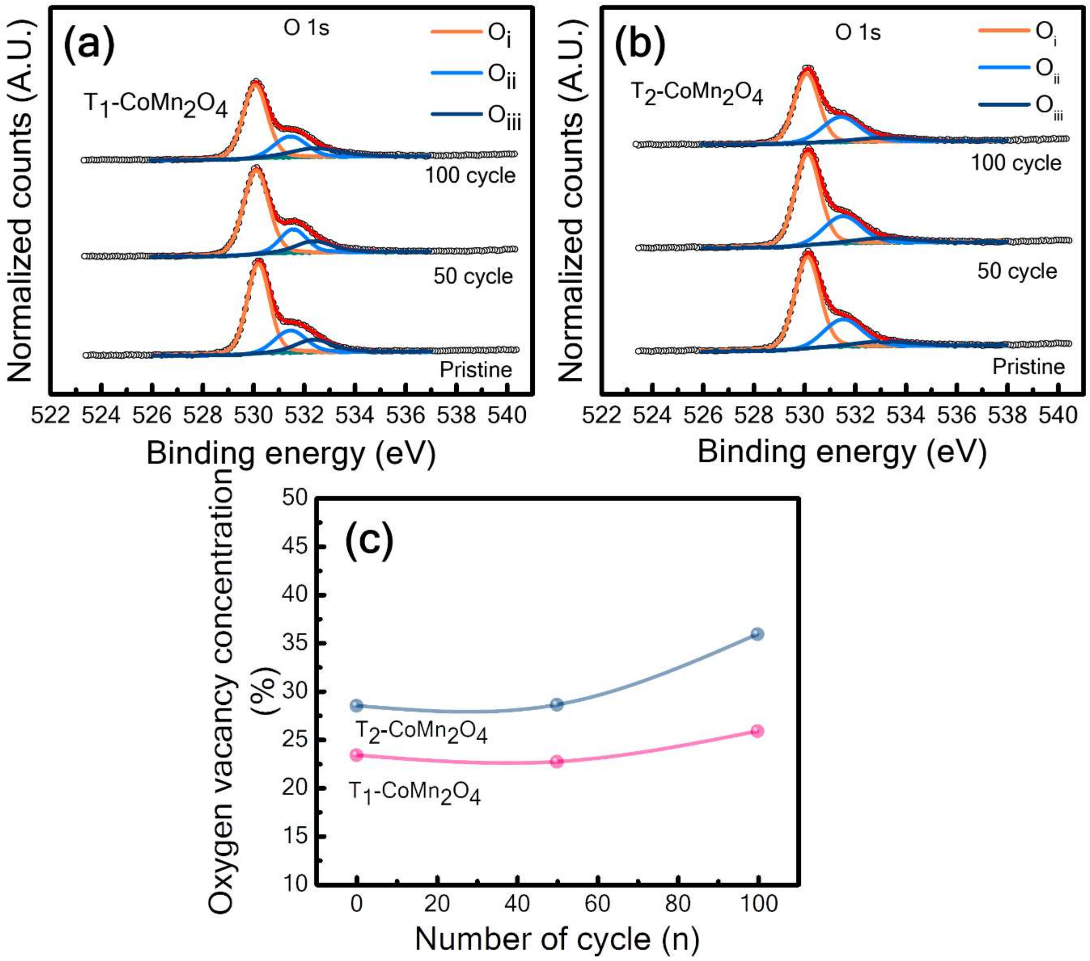

Figure S2 (a) and (b) Ex situ XPS studies of O1s of T_1-$CoMn_2O_4$; and T_2-$CoMn_2O_4$, respectively, in the cathode of the supercapacitor containing active material (T_1 or T_2), conductive material (carbon black) and binder (PVDF) and (c) Variation of oxygen vacancies concentration of T_1-$CoMn_2O_4$and T_2-$CoMn_2O_4$ in the cathodes after different consecutive CV cycles.

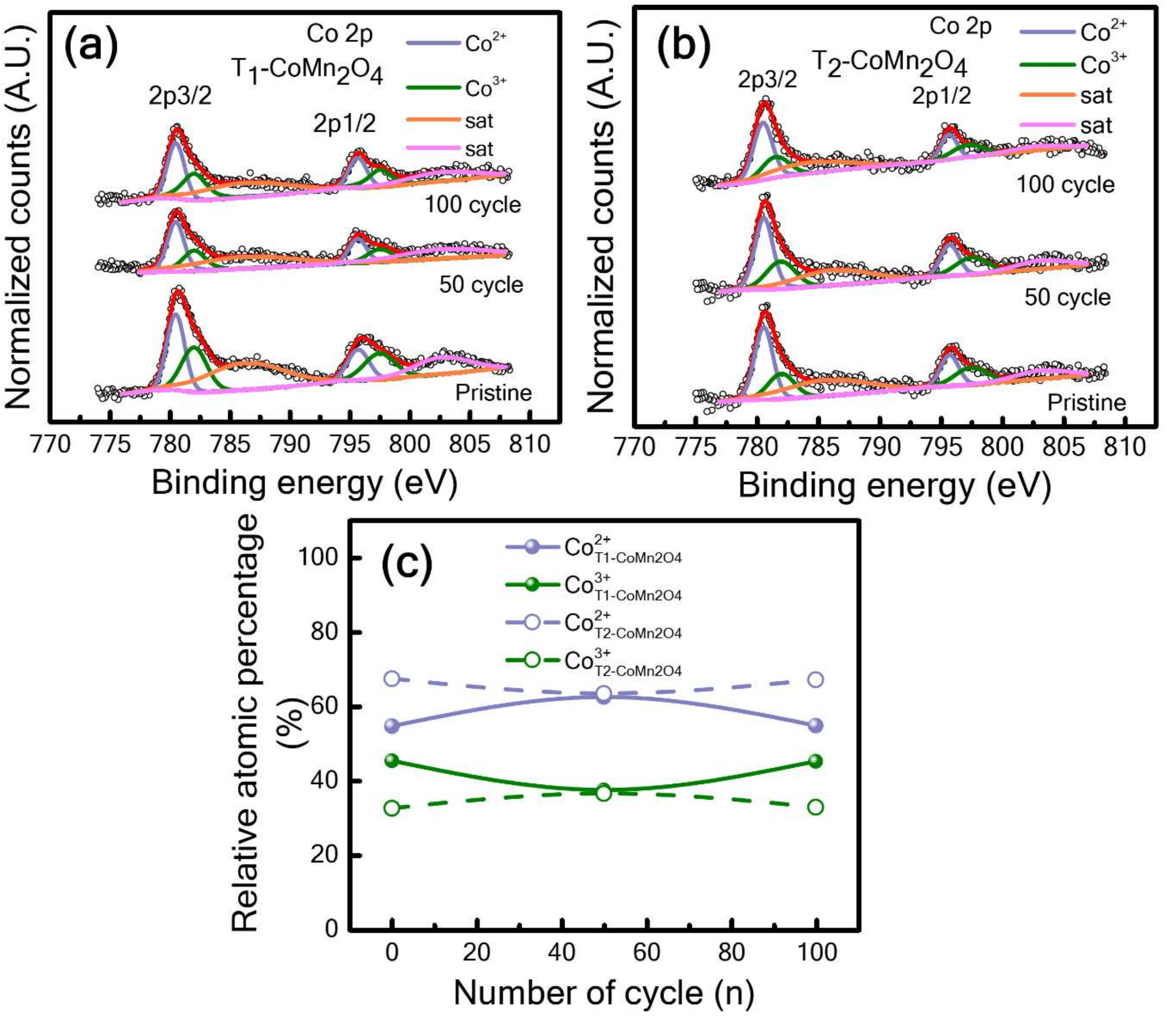

Figure S3 (a) and (b) Ex situ XPS studies of Co 2p of T_1-$CoMn_2O_4$ and T_2-$CoMn_2O_4$, respectively, in the cathode containing active material (T_1 or T_2), conductive material (carbon black) and binder (PVDF) of the supercapacitor and (c) Variation of oxidation changes of Co^{2+} and $Co^{3+}$of T_1-$CoMn_2O_4$ and T_2-$CoMn_2O_4$ in the cathodes after different consecutive CV cycles.
